# Breast Carcinoma – A Comparative Study of Immunohistochemistry and Fluorescence in Situ Hybridization for Her-2 Assessment and Association of ER, PR, HER-2 and Ki-67 Expression with Clinico-Pathological Parameters

**DOI:** 10.30699/IJP.2022.538167.2712

**Published:** 2022-09-02

**Authors:** Sajitha K, Meenakshi Arumugam, Jayaprakash Shetty, Reshma A Shetty, Ritu Asani, Prashanth D Shetty

**Affiliations:** 1 *Department of Pathology, K S Hegde Medical Academy, NITTE (Deemed to be University), Deralakatte, Mangalore, Karnataka, India*; 2 *KSHEMA Centre for Genetic Services, K S Hegde Medical Academy NITTE (Deemed to be University), Deralakatte, Mangalore, Karnataka, India*

**Keywords:** Carcinoma, Immunohistochemistry, In situ Hybridization, Fluorescence, Triple Negative breast neoplasms

## Abstract

**Background & Objective:**

Breast cancer is the most common cancer in developed and developing countries. This study mainly addresses the issue of an equivocal result in IHC, which then needs further assessment if the patient has to receive targeted therapy. The study aimed to detect the expression of Her2/neu protein in breast cancer by immunohistochemistry (IHC) and Fluorescence in situ Hybridization (FISH) and evaluate concordance and discordance between the two methods. Also, the clinicopathological parameters in these patients were studied in association with ER, PR, HER-2, and Ki-67.

**Methods:**

This study was conducted on 34 female carcinoma breast specimens, including core biopsies and mastectomies. Each case underwent histopathological and immunohistochemical studies for (Estrogen Receptor) ER, (Progesterone Receptor) PR, (Human Epidermal growth factor Receptor 2) HER-2, and Ki-67. In addition, FISH was done on all the samples to detect *Her2* gene amplification.

**Results:**

The overall concordance between the two tests was 79.41% while the concordance between the two tests in equivocal cases, was 14.3%. ER/PR expression and HER-2 amplification were inversely associated. Also, Ki-67 expression was not associated with the side size of the lesion, lymphovascular invasion, and lymph node metastasis. Age less than 50 at presentation and infiltrating ductal carcinoma histological type showed increased proliferation index.

**Conclusion:**

The highest concordance between FISH and IHC was noted in IHC positive and negative cases, whereas IHC equivocal cases showed low concordance. FISH accurately determines the assessment of HER2 expressions in equivocal cases.

## Introduction

Breast cancer is common cancer in developed and developing countries. The projected incidence of Breast cancer in 2020 in women is 2,05,424 and 5377 men making 1 out of 29 women and 1 out of 1022 men susceptible to breast cancer in the Indian population (1). The diagnosis and management of breast cancer need a multidisciplinary approach to reduce recurrence and disease-associated mortality and morbidity. Breast Carcinoma is a heterogeneous tumor with various histological subtypes, clinical, pathological, molecular features, and variable prognosis. The advent of molecular biology has made tumor subtyping, predicting metastasis, and prognostication more authentic with improved treatment algorithms.

Detection of tissue biomarkers like Estrogen Receptor (ER), and Progesterone Receptor (PR) predicts response to therapy and post relapse survival. Estrogen plays a significant role in developing breast cancer, and carcinoma is associated with consistent exposure of breast epithelium to estrogen. Firstly, estrogen binding stimulates the cell cycle, which increases the chances of replication errors, and secondly, the toxic substances produced during estrogen metabolism damage the DNA. Both processes initiate and promote the process of tumorigenesis. Estrogen receptor-positive breast cancers are responsive to hormone therapy and have a good prognosis (2). Estrogen has an essential role in inducing progesterone receptors. 

Another biomarker tested along is HER-2/neu oncogene (also known as c-erbB-2). This gene is a member of the ErbB family of receptor tyrosine kinases, located on the long arm of chromosome 17 (17q12–21.32), and codes for HER 2 proteins. Studies have shown that 15-20% of breast cancer cases with HER-2/neu overexpression are associated with poor prognoses (3). Trastuzumab, a monoclonal antibody, is targeted against the HER-2/neu gene, but its use demands the accurate expression of HER-2 protein, which can be done by immunohistochemistry (IHC) and Fluorescence in situ Hybridization (FISH). IHC is the less expensive method with high sensitivity and moderate specificity and assesses the protein expression with the help of antibodies (4). Proliferative markers like Ki-67 are now known to be prognostic markers as well. Studies show an association of Ki-67 with tumor grade. The most common method to measure Ki-67 is IHC by MIB-1 antibody (5).

This study aims to determine the status of HER2/neu in breast carcinoma tissue specimens by immunohisto-chemical and FISH techniques and the association of clinicopathological parameters with ER, PR, HER-2/neu, and Ki-67.

## Material and Methods

**Study Design and Setting**

This descriptive study was carried out in the department of pathology and KSHEMA Centre for Genetic Services, KS Hegde Medical Academy, Mangalore, India, from November 2018 to January 2020. Thirty-four tissue samples from breast carcinoma specimens, including core biopsies and mastectomy, were obtained for IHC and FISH. The core biopsies were then followed up for further data regarding the morphological features, grade, and tumor stage during mastectomy. The clinical and demographic characteristics were obtained from the case records. IHC was not repeated in any of the mastectomies. All surgical specimens from patients with a clinical diagnosis of carcinoma breast were included in this study. Patients with recurrent tumors and those given neoadjuvant chemotherapy were excluded.

**Histopathologic Studies**

The histopathological diagnosis of breast carcinoma was established by standard light-microscopic (Olympus, Japan) evaluation of paraffin sections stained with Hematoxylin and Eosin in each case and graded based on Nottingham modification of the Bloom–Richardson score. 

**IHC Analysis**

Estrogen and progesterone receptor (ER, PR), HER-2/neu, and Ki-67 analyses were done by immune-histochemical analysis as a part of the routine work-up of all the tumors. The immunostaining was done on the 3 -5 µ thick sections. Antigen retrieval of deparaffinized and rehydrated samples was done in citrate buffer (pH 6.0) in a microwave oven for 8 min. After cooling to room temperature, endogenous peroxidase blockage was performed using 3% (w/w) hydrogen peroxide. After sample washing (Tris buffer, pH 7.6), primary estrogen receptor alpha (Clone EP1), Progesterone Receptor (Clone EP 2), HER2/ ErbB2 (Clone EP 3) (Mouse and Rabbit Monoclonal Antibody, PathnSitu Biotech-nologies, India), antibodies were applied for 60 min at room temperature. Visualization was achieved by incubating slides with Poly Excel HRP labeled polymer for 10 minutes and diaminobenzidine (DAB) for 10 minutes, followed by washing and counterstaining with Mayer's hematoxylin. Ran an external positive control with every batch and scoring; the immunoreactivity of the tumor cells was determined by visual estimation and quantitation using the all-red scoring system. The total score was given as a sum of the percentage of positive cells (scored as 0 – 5) and the staining intensity of nuclei (scored as 0 – 3). A sample is considered ER or PR negative if < 1% or 0% of tumor cell nuclei are immunoreactive according to the guidelines of the American Society of Clinical Oncology (ASCO)/ College of American Pathologists (CAP) (6).

HER-2/neu evaluation was done in a semi-quantitative manner based on the membrane positivity according to the guidelines of ASCO/ CAP (6), and the results were graded as follows: 0, 1+, 2+, and 3+. Grade3+ was taken as positive, 0/1+ as negative, and 2+ as undetermined/equivocal. Ki-67 - Immunostaining for Ki-67 was done, and the nuclear stain was assessed as the number of positively stained tumor cells among the total number of neoplastic cells and then categorized into three groups – as low (<15%), moderate (15 – 30%) and high (>30%) (3,7,8) Based on IHC, the tumors were classified as Luminal A (ER and PR positive HER-2 negative Ki-67 'low'), Luminal B (ER-positive HER-2 negative and Ki-67 'high) or (ER-positive HER-2 over-expressed or amplified, Any Ki-67), basal-like (ER and PR absent, HER-2 negative), HER-2/neu + (HER-2 over-expressed or amplified ER and PR absent).

**FISH Analysis**

FISH HER-2/neu amplification was done on all samples using parallel sections from the same tissue block using XL ERBB2 (HER-2/neu) amplification probe kit (Metasystems probes, Germany). The slides were deparaffinized at 60ºC using xylene 1 for 15 min, followed by xylene 2 for 15 min, followed by dehydration in graded series of 70%, 85%, 95%, and 100% ethanol for 5 minutes each at room temperature. Slides were air-dried at room temperature. Pre-treatment of the slide was done using an Aquarius Tissue pre-treatment kit (Metasystems probes, Germany), and the procedure was done based on the manufacturer's protocol. One hundred nuclei were analyzed to detect the HER-2/neu amplification, and cases were scored according to ASCO/CAP criteria. Results were considered FISH negative when two red (HER-2) and two green signals (CEP 17) in interphase cells were seen. Amplification of the HER-2/neu locus showed multiple red signals (HER-2/neu/CEP17 ratio of 2 or more) and was considered as FISH HER-2/neu positive. Good, amplified signals and cells were captured using an Olympus BX53 fluorescence microscope equipped with DAPI, FITC, TEXAS Red filters, and FISH View image acquisition (GENASIS – Version 8.1.1, Applied Spectral Imaging, Germany).

**Statistical Analysis**

Data were analyzed and recorded as frequency and percentage. The relationship between the various clinical and histopathological parameters and statistical analysis for correlation among these parameters was determined using the Pearson chi-square test. Significance was assumed at P-value<0.05. Data management and analysis were performed by using Microsoft Excel and Statistical Package for the Social Sciences v19.0.1 (SPSS Inc., Chicago, IL., USA). 

## Results

A total of 34 breast tissue samples from female patients, including core biopsies and mastectomy specimens, were analyzed in this study. Out of these, 17 (50%) were trucut, and 17 (50%) were mastectomy specimens. The trucut biopsies were followed up for the data regarding staging during mastectomy.

Patients belonged to an age range of 31-80 years with a mean age of 50.23 years ±12.56. The frequency was nearly identical in both breasts, with 16 (47.05%) patients having a lesion in the right breast and 18 (52.95%) in the left breast. Tumor size was between 2-5 cm in 19 (55.88%) patients, 09 (26.47%) cases had tumor size less than 2 cm, and 06 (17.65%) cases more than 5 cm. 

**Fig. 1:**
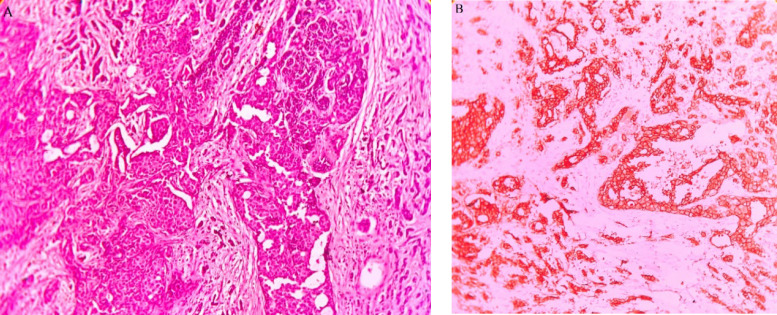
a: Invasive carcinoma breast, NOS. H & E x 100. b: Positive Her-2 testing by IHC shows complete circumferential membrane staining in >10% of tumoral cells

Patients diagnosed to have infiltrating ductal carcinoma (Figure 1a) were 20 (58.82%), those with infiltrating lobular carcinoma were 13 (38.24%), and one case (2.94%) was diagnosed with Metaplastic carcinoma. Histological grading by Nottingham modification of Bloom Richardson system showed grade II tumor in 16 (47.06%) cases, followed by grade III in 13 (38.24%) and grade I in 5 (14.71%) cases. The majority of the patients had tumor stage III with 19 (55.88%) cases, tumor stage I was seen in 06 (17.65%) and stage II in 09 (26.47%). Lymphovascular invasion was seen in 13 (38.24%) cases, and lymph node metastasis was present in 21 (61.76%).

**Fig. 2:**
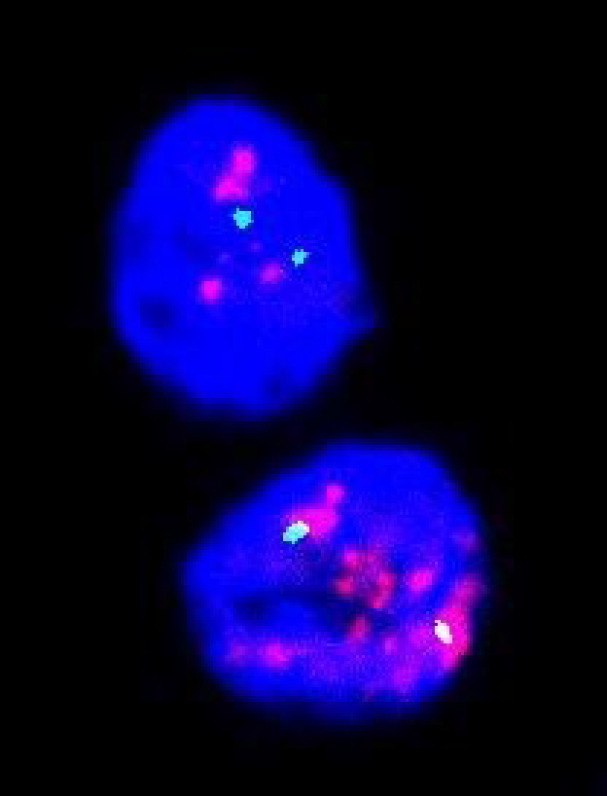
FISH image showing positive results for HER-2/neu gene amplification: red signals in clusters of 8–10 and 2 green signals per nucleus

**Fig. 3:**
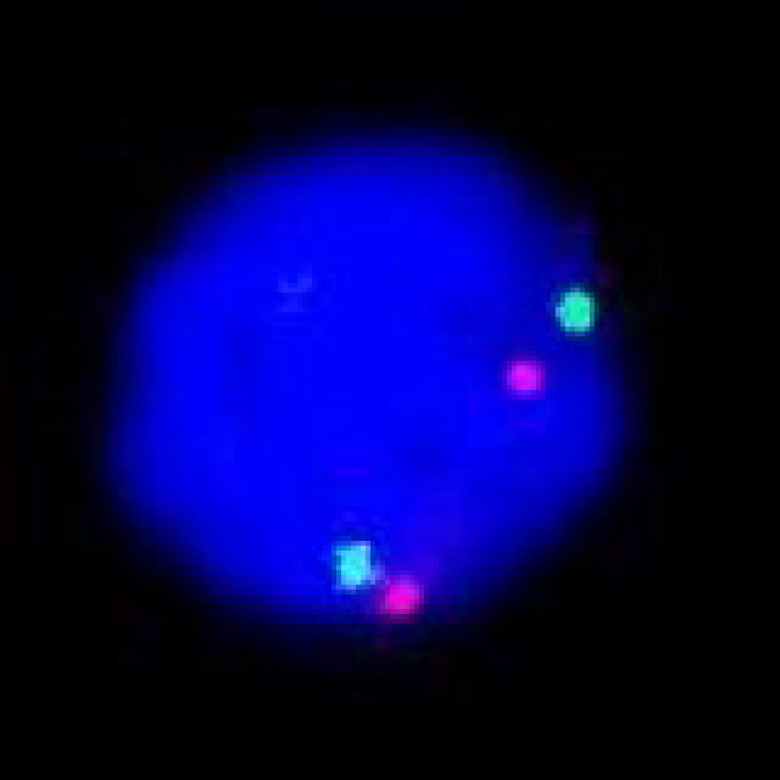
FISH image showing negative results for HER-2/neu gene amplification: 2 red signals and 2 green signals per nucleus

ER status analysis showed 13 (38.24%) patients as ER-positive, and 21 (61.76%) were ER-negative. PR positive status was seen in 11 (32.35%), and PR negative was 23 (67.65%). Ten (29.41%) cases assessed by IHC for HER-2 showed positivity (Figure 1b), the majority, i.e., 17 (50%), were negative, and 07 (20.59%) were equivocal. On analysis by the FISH technique, all IHC positive showed amplification (Figure 2), and IHC negative cases were not amplified (Figure 3). Among the equivocal cases, one case was found to be HER-2/neu amplified, while the remaining 6 cases were not amplified.

HER-2 status and Ki-67 labeling index were correlated with the various clinicopathological parameters (Table 1). No significant statistical association was seen between HER-2 status and age, tumor size, lymph node infiltration, and tumor grade. A majority of grade 2,3 tumors did not show amplification. The most frequent breast cancer subtype in the studied patients was Luminal in 15 cases (44.12%), followed by Basal-like or Triple-negative breast cancers (TNBC) in 13 cases (38.23%) and HER-2/neu in 6 cases (17.65 %). In our study among patients aged under 55, 4 (20.8%) cases were Luminal A (ER + or PR + or both, HER-2/neu -), 6 (16.7%) were Luminal B (ER + or PR + or both, HER-2/neu +) or (ER+, low PR+, HER-2/neu -, high Ki67), 9 (37.5%) cases were TNBC (ER−, PR−, HER-2/neu -) and 5 (25%) cases were HER-2/neu + (ER−, PR−, HER-2/neu +). Among patients aged above 55 years, the incidence was 3 (30%) - Luminal A, 2 (20%) - Luminal B, 4 (40%) – TNBC, and 1 (10%) – HER-2/neu +.

The mean value of Ki-67 was low in 15 cases (44.1%), intermediate in 6 patients (17.64%), and high in 13 cases (17.65%). Infiltrating ductal carcinomas showed a greater incidence of high Ki-67 than invasive lobular carcinomas. There was no statistical significance between Ki-67 and tumor size, stage and grade of tumor, ER/PR and Her2/Neu status, lymphovascular invasion, and lymph node metastasis.

The concordance between the results of the two tests was 100% each for IHC negative and IHC positive cases, and the concordance was 14.3% in the equivocal cases (Table 2). A total concordance of 79.41% was noted, defined as IHC 2+ / 3+ and HER-2/neu FISH positive, or IHC 0 / 1+ and HER-2/neu FISH negative. Discordance is the discrepancy between the IHC and HER-2/neu FISH, defined as IHC 2+ or 3+ but HER-2/neu FISH negative or IHC 0 or 1+ but HER-2/neu FISH positive. The discordance rate was 0 % among the IHC positive (3+) and IHC negative (0 / 1+) cases. The discordance rate in the IHC 2+ (equivocal) cases was 85.71%. The overall discordance rate by IHC was 17.64%. 

**Table 1:** Association of HER-2/neu status with clinical findings, ER, PR status, histological parameters, and Ki-67 labelling index

Parameters	*HER-2/neu* FISH				Ki 67			
	Amplified	Non-Amplified	Inconclusive	P-value	Low (≤15 %)	Intermediate (16 – 30%)	High >30%	P-value
Age ≤ 55 24 > 55 10	08 02	15 08	01 -	0.774	10 05	04 02	10 03	0.954
T size (cm) <2 09 2-5 19 >5 06	05 03 02	03 16 04	01 - -	0.054	05 07 03	01 03 02	03 09 01	0.585
Histologic grade I 05 II 16 III 13	02 04 04	03 11 09	- 01 -	0.853	02 08 05	01 01 04	02 07 04	0.554
Regional Lymph nodes Present 21 Absent 13	05 05	16 07	- 01	0.282	08 07	06 -	07 06	0.104
ER status Positive 13 Negative 21	04 06	09 14	- 01	0.962	05 10	02 04	06 07	0.756
PR Status Positive 11 Negative 23	04 06	07 16	- 01	0.592	06 09	01 04	04 09	0.691
Hormonal Receptor Status ER +, PR + 09 ER +, PR - 04 ER -, PR + 02 ER -, PR - 19	-	-	-	-	04 01 02 08	01 01 - 04	04 02 - 07	0.731
*HER-2/neu* FISH Amplified 10 Non-Amplified 23 Inconclusive 01	-	-	-	-	03 12 01	01 05 -	06 06 -	0.319

**Table 2:** Comparison of *HER-2/neu* amplification results by IHC and FISH

IHC scoring	*HER-2/neu* /FISH positive	*HER-2/neu* /FISH negative	Concordance by IHC	Discordance by IHC
0/1+ (n=17)	0	17	(17/17) 100%	(0/17) 0%
2+ (n=7)	1	6	(1/6) 14.3%	(6/7) 85.7%
3+ (n=9)	9	0	(9/9) 100%	(0/9) 0%

## Discussion

Breast cancer is the most common cancer in India, with the highest number of new cancer incidences per year (14%) and with a high incidences-to-mortality ratio (approximately 50%) (9). An annual rise of 0.5%–2% in the incidence, with a more significant increase, was noted in females less than 45 years (10).

 Our study was done on 34 patients, and the median age was 48.5, with a mean age range of 50.23 ±12.56. Of these, 24 (70.6 %) patients were ≤55 years of age. Other studies have also reported a similar mean age range (11,12,13). A large retrospective study of 5436 women from a regional cancer center in South India found the median age to be 48 years, and 65% were ≤55 years (14).

With the increased use of targeted therapies, diagnosis and classification of cancers are based more often on molecular profiles and immunohistochemistry. The breast cancer classification based on molecular profiling by DNA microarray analysis divides it into six molecular subtypes. The corresponding categories based on IHC include Luminal A (ER and PR positive HER-2 negative Ki-67 'low'), Luminal B (ER-positive, HER-2 negative and Ki-67 'high) or (ER-positive HER-2 over-expressed or amplified, Any Ki-67), basal-like (ER and PR absent, HER-2 negative), HER-2/neu + (HER-2 over-expressed or amplified ER and PR absent) (15). It is recommended to report any degree of estrogen receptor positivity so that the patient can benefit from the endocrine therapy. Most of our tumors were hormone receptor-positive (44.12%). This showed a higher incidence (29.41%) under 56 years of age compared to that above the age of 55 years (14.71%). Other studies have also shown comparable incidences of hormone receptor positivity with 48% (14), 49% (16), and 49.2% (17).

Earlier studies have shown a greater incidence of ER-negative breast cancers in India than in the west, and this was thought to be due to variations in the technical aspects in staining and reporting, younger age of patients, and advanced stage at the time of clinical presentation (18). More recently, however, with automation and better pre-analytical handling of tissues, there has been an increase in hormone-positive tumors. According to the revised ASCO-CAP guidelines 2010, the threshold for ER positivity has been lowered to 1%, which has also contributed to a decrease in ER-negative cases. Studies have reported an increase in the incidence of ER negativity in younger patients (14,17). In a large study from south India, a greater incidence of ER positivity was found in premenopausal (56.1%) as compared to postmenopausal women (47.4%). (10) Of the 21 patients who were ER-negative in our study (two were PR positive), 15 (71.43%) patients were ≤55 years, and 6 (28.57%) patients were >55years.

The triple-negative cancers (TNBC) are also referred to as 'basal-like because their gene expression profile resembles that commonly expressed in basally located myoepithelial cells in the ducts. They are more likely to present as a palpable mass and have a more aggressive clinical presentation with an increased frequency of recurrence (19). The incidence of TNBC in the western population is 12.2% – 13% of all breast carcinomas, with a higher incidence in blacks and among Indian Asian women (15.4%) (20). In a meta-analysis of 34 studies of TNBC in India, a higher prevalence of TNBC was noted with a pooled prevalence of 27% (95% CI, 24% to 31%) (9). The incidence of TNBC was 13 (38.23%) cases in our study, and nine patients (69.23%) were of the age ≤55years. One study reported a lower incidence (24.8%) of TNBC with a greater incidence of ER-negative and TNBC among women younger than 50 (17).

Kulkarni *et al.*, found that irrespective of their variations in prevalence, the TNBC are high grade and more common in younger women with greater odds of presenting with lymph node positivity (9). Lymph node positivity in our study was noted among 10 (77%) out of 13 TNBC. 

Overexpression of HER-2 was seen in 25-30% of invasive breast cancers (21). The incidence of HER-2 positive cases in the Indian subcontinent is similar to that described in the Western literature (15). The number of HER-2 positive and negative hormone cases in our study was 6 (17.65%). Our study reported no association between age and HER-2 expression.

 Lymph node staging plays a significant role in tumor staging and deciding treatment plans and prognoses. The survival rate decreases with more lymph nodes showing metastasis. In the present study, tumor grade and lymph node metastases did not show any association with HER-2/neu gene amplification, as noted in other studies (23,24). A few authors have shown a significant association between the two (25,26). The present study shows an inverse association between ER, PR, and HER-2 gene amplification but did not find effective statistical results (*P*>0.05). This can be attributed to the small sample size. Literature shows a significant association with ER, PR being inversely related to HER-2 expression (12,23,25).

Ki-67 is a cellular marker for proliferation and is widely used as an additional marker for deciding on adjuvant chemotherapy (27). In this study, all 34 patients underwent a Ki-67 labeling index. There was an increased expression of Ki-67 in infiltrating ductal carcinoma type. This was compatible with other studies (11,27). We could not demonstrate any statistical association between Ki-67 proliferation index and side, lesion size, lymphovascular invasion, lymph node metastasis, hormone receptor status, and tumor grade. 

 This study also compared the results of IHC and FISH in assessing HER-2/neu status of tumors. Clinically recommended methods include IHC and ISH methods using fluorophores, chromogens, or silver preparations. Both tests show a high level of correlation in the evaluation of HER-2/neu status using formalin-fixed paraffin-embedded specimens (28). IHC is a relatively cheap and fast method, while FISH is technically more demanding, time-consuming, and expensive (29).

Most of our patients (50%) were classified as HER-2 negative (IHC 0/1+), 29.41 % were HER-2 positive (IHC 3+), and 20.6% were classified as equivocal (IHC 2+). Successful Hybridization was seen in 33 (97%) cases. One sample which tested positive (IHC3+) with IHC failed to show Hybridization with the FISH test; the FISH failure rate was 2.9%. This was probably due to the depletion of tumor tissue in the block following IHC as it was a trucut biopsy specimen. Of 10 HER-2/neu FISH amplified cases, 9 (90%) were scored IHC 3+, 1 (10%) was scored IHC 2+ and none was scored IHC 1+ or 0. Among the 23 FISH-negative cases, 6 (26.1%) had an IHC score of 2+, and all the other samples, 17 (73.9%), were negative. The two tests showed high concordance in the IHC negative (100%) and IHC positive (100%) cases but low concordance in the equivocal cases (14.3%). Various studies show a concordance rate between IHC and FISH ranging from 79% to 100% for IHC 3+ cases (23,24) and between 12% to 36% for IHC 2+ cases (4, 21). 

The discordance rate for IHC 2+ cases in our study was 85.7%. In their study, Payandeh *et al*., studied samples from 133 patients for detection of HER-2/neu in IHC 2+ and 3+ cases and found a high concordance rate (81.25%) for IHC 3+ cases with a high discordance rate in the IHC 2+ (67.6%) (13). In another study, 30 paraffin-embedded tissue blocks from 73 breast cancer patients were used, and the correlation between the IHC and FISH HER-2/neu was evaluated. Concordance rate was 100%, 18.18% and 83.33% for IHC 0/1+, 2+ and 3+ groups, respectively. Total concordance was 84.72%. Various studies have reported a high discordant rate between the tests in IHC 2+ cases (13,29,30,31) which was observed even in our study. This may be due to the variability in the interpretation during IHC reporting, variations in the pre-analytic conditions, and imperfect IHC techniques. Other studies have reported variability in the sensitivity and specificity of antibodies and probes used and chromosome 17 polysomy, resulting in discrepancies between protein expression and gene amplification. 

Although cost and turnaround time was greater for FISH, patients with tumor scores interpreted as 2+ by IHC would benefit from FISH to assess HER-2/neu more accurately status and avoid inaccurate prognostication and inappropriate treatment (31). IHC can be used initially as a screening test for detecting HER-2/neu, and FISH is used to determine gene amplification, especially in equivocal cases (13,21,23,30). The small sample size decreases the statistical power of this study. The sample size was limited due to economic constraints. A similar study needs to be carried out on a larger population of patients to validate the findings.

## Conclusion

There was no statistically significant association between hormone receptor status and HER-2/neu amplification. We also could not demonstrate any significant association between the Ki-67 and various clinicopathological parameters. As a result of the high discordance in the equivocal cases, it is recommended to perform a FISH analysis to detect HER-2/neu in all cases of IHC score 2+. Accurate assessment of the gene amplification by FISH, especially in equivocal cases, will help identify the target patients who can benefit from Trastuzumab's therapeutic effects. 

##  Ethics approval & Consent to Participate

Ethics approval was taken from Institutional ethics committee and consent form was taken from all the participants.

##  Authors' contributions

SK: Study conception and design, IHC analysis and interpretation; MA: Data collection; MA & RAS: Analysis and interpretation of FISH results; RA: Initial drafting of manuscript; JS & PDS: Revised it critically for important intellectual content. 

## Conflict of Interest

The authors declared no conflict of interest.

##  Funding

The author(s) received financial support from NITTE (Deemed to be University) for conducting the research.
